# Editorial: Diversity of beetles and associated microorganisms

**DOI:** 10.3389/fmicb.2023.1252736

**Published:** 2023-07-26

**Authors:** Hassan Salem, Peter H. W. Biedermann, Takema Fukatsu

**Affiliations:** ^1^Mutualisms Research Group, Max Planck Institute for Biology, Tübingen, Germany; ^2^Chair of Forest Entomology and Protection, University of Freiburg, Freiburg im Breisgau, Germany; ^3^Bioproduction Research Institute, National Institute of Advanced Industrial Science and Technology, Tsukuba, Japan; ^4^Department of Biological Sciences, Graduate School of Science, The University of Tokyo, Tokyo, Japan; ^5^Graduate School of Life and Environmental Sciences, University of Tsukuba, Tsukuba, Japan

**Keywords:** Coleoptera, insects, beetles, endosymbionts, gut symbionts, ectosymbionts, bacteriocytes

Symbioses with beneficial microbes serve as a source of evolutionary innovation across numerous insect clades (Moran, 2007; Douglas, 2015). Beetles, representing the most speciose insect order Coleoptera, rely on symbioses for numerous adaptations (Biedermann and Vega, 2020; Salem and Kaltenpoth, 2022). From upgrading the nutritional physiology of herbivorous taxa (Biedermann and Taborsky, 2011; Vigneron et al., 2014; Ceja-Navarro et al., 2015; Anbutsu et al., 2017; Hirota et al., 2017; Vogel et al., 2017; Salem et al., 2020), to endowing defensive traits to fend off antagonistic threats from predators and pathogens (Piel, 2002; Flórez et al., 2017; Berasategui et al., 2022), microbial symbioses are a key feature behind the evolutionary success of beetles. This Research Topic aimed to shed light on the diversity and functional aspects of beetle-microbe interactions, spanning the coleopteran tree of life. Notably, we aimed to highlight the role of molecular and analytical advancements in fueling research into how these partnerships are maintained and transmitted, their impact on beetle metabolism and physiology, and finally, their influence on ecological interactions and how beetles adapt to their environment.

The Research Topic attracted articles examining bacterial symbiont diversity, colonization, localization, and transmission across several beetle clades. These featured surveys of the general gut microbiome of several taxa, including the wood-borer beetle *Agrilus mali* (Buprestidae) (Bozorov et al.). The beetle hosts a stable bacterial community but appears to lack a persistent fungal one. In clarifying whether the gut bacterial community actively excludes plant-associated fungi, the authors applied several analytical techniques to highlight the inhibitory effects of bacteria-produced compounds. Prior exposure to pathogens can also shape the bacterial community associated with beetles, as demonstrated in the red flour beetle *Tribolium castaneum* (Tenebrionidae), and highlighting an interplay between the host immune system with some members of the resident microbiome (Korša et al.). Predacious insects such as the ladybird beetle *Harmonia axyridis* (Coccinellidae) can similarly host a diversity of bacterial associates, including *Staphylococcus, Enterobacter, Glutamicibacter*, and *Acinetobacter* (Du et al.). But how variable is this community throughout the developmental cycle of its holometabolous host? These taxa varied considerably in abundance between adults and larvae, suggesting stage-specific roles in the beetle host (Du et al.).

The Research Topic featured additional contributions examining the role of the gut microbiome in facilitating herbivory in beetles, including the Asian longhorned beetle *Anoplophora glabripennis* (Cerambycidae) (Wang et al.), the coffee berry borer *Hypothenemus hampei* (Curculionidae: Scolytinae) (Vega et al.), and tree sap beetles *Nosodendron* spp. (Nosodendridae) (Hirota et al.). As a wood-boring insect, *A. glabripennis* must contend with a diet rich in complex plant polymers. The authors detected a microbial community dominated by *Enterococcus, Gibbsiella, Wolbachia*, and the ascomycete *Fusarium*, and inferred their functional roles involved in the degradation of lignocellulose, detoxification of noxious plant metabolites, and fixing nitrogen that may be limiting in the beetle's diet. For the coffee berry borer, previous studies implicated that the beetle's gut microbiome is involved in detoxification of caffeine within the coffee seed (Ceja-Navarro et al., 2015). Here, through a cultivation-based approach, Vega et al. pinpointed members of the beetle's bacterial community encoding detoxification genes that may underlie caffeine breakdown, including *Acinetobacter, Bacillus, Pseudomonas*, and *Stenotrophomonas*. In tree sap beetles, Hirota et al. revisited early histological descriptions to characterize the endosymbiont associated with *Nosodendron coenosum* and *Nosodendron asiaticum* using molecular, phylogenetic, and histological approaches. The study revealed a Bacteroidota symbiont that inhabits the bacteriocytes within the host beetles and appears to be transmitted via ovarial passage. The authors posited a potential nutritional role given the phylogenetic position of the symbiont relative to other beetle-associated microbes.

Faithful transmission of beneficial symbionts is a common characteristic of stable insect-bacterial symbioses (Bright and Bulgheresi, 2010; Salem et al., 2015). Transmission can be vertical, either through the germline (Koga et al., 2012; Luan et al., 2016) or via maternal secretions (Kaiwa et al., 2014; Pons et al., 2022). Alternatively, insects can horizontally acquire their symbionts every generation from the environment (Kikuchi et al., 2007). Additional support for the latter is included in this Research Topic (Paddock et al.; Avila-Arias et al.; Wierz et al.). While the corn rootworm (Chrysomelidae) shared some members of their bacterial communities across geographic populations, variation within the host species appeared to correlate with the geographical distance between sampling sites, implicating environmental effects in shaping the insect's microbiome (Paddock et al.). A similar case was reported for the Japanese beetle *Popillia japonica* (Scarabaeidae), whose larvae acquire their fungal gut symbionts necessary for cellulose degradation from the environmental soils (Avila-Arias et al.). The darkling beetles *Lagria* spp. (Tenebrionidae) rely on the antibiotic-abilities of their *Burkholderia* (= *Caballeronia*) symbionts (Flórez et al., 2017, 2018). While these symbionts are vertically transmitted through maternal secretions on surfaces of newly laid eggs, Wierz et al. explored whether the different symbionts can additionally be acquired from the beetle's environment. Despite a transition to an insect-associated lifestyle, *Burkholderia* retained the ability to proliferate within plants, highlighting the likely role of mixed-mode transmission for the persistence of a defensive ectosymbiont (Wierz et al.).

While bacterial symbioses dominate in many insect taxa, beetles are commonly associated with fungal symbionts, in particular those feeding on woody materials (Buchner, 1928, 1965; Biedermann and Vega, 2020). In this Research Topic, notably, around two-thirds of all the articles are featuring fungal symbioses, among which the majority dealt with wood-feeding beetles. Almost half of all the articles addressed the fungal symbioses of bark and ambrosia beetles (Curculionidae: Scolytinae), a group currently studied intensively due to their emerging importance as (invasive) pests in forestry and agriculture (Hulcr and Stelinski, 2017; Biedermann et al., 2019). Xylem-boring ambrosia beetles live in obligate mutualism with fungi that they grow for food in their tunnels, while in phloem-feeding bark beetles, their fungal symbionts are often facultative and their overall role in nutrition, detoxification and tree killing is still under debate (Six and Wingfield, 2011). What nutritional roles underpin the importance of the fungi for the host ambrosia beetles was impressively demonstrated by Lehenberger, Foh et al.. By the joint application of SEM-EDX and ecological stoichiometry to an animal-microbe mutualism, they visualized the fungal translocation of micronutrients (in particular nitrogen and phosphorus) from the wood substrate and strong concentration of them within the fungal tissues exploited by their beetle hosts. Such nutritional upgrading may also be present but not essential in bark beetles, given that their phloem substrate is nutrient-richer *per se*. The vast variety of the beneficial effects of the fungi for specific bark and ambrosia beetles was reviewed by Six and Klepzig. They showed that beneficial effects of fungi to species in this group are strongly dependent on ecological aspects of the beetle species, which may be even context-dependent in species with facultative symbionts. This possibility has been overlooked so far, but may partly explain the lack of symbionts like *Endoconidiophora polonica* in the European Spruce bark beetle *Ips typographus* (Curculionidae: Scolytinae) in some populations, although the symbionts were determined as highly beneficial in other populations (Kirisits, 2004).

Benefits to insects may be just by-products of normal fungal physiology as in facultative and environmentally acquired mutualists of the Japanese beetle *Popillia japonica* (Scarabaeidae) involved in cellulose degradation (Avila-Arias et al.), or elaborate and specific as in obligate mutualists of wood-dwelling stag beetles (Lucanidae) and ambrosia beetles (Curculionidae: Scotylinae) that are highly dependent on their fungal associates. Such mutualisms are characterized by vertical transmission of the fungal associates using specialized organs called mycangia or mycetangia (e.g., Mayers et al., 2022). Given these close associations, it is not surprising that the mutualists may restrict the ecological niche of their hosts, as shown for the heat tolerance of *Platycerus* stag beetles (Lucanidae) determined by associated *Scheffersomyces* yeast symbionts (Zhu et al.). Similarly, the fungal symbionts of bark and ambrosia beetles determine tolerance against and attraction to ethanol, which is released under high plant stress and commonly present in xylem (Lehenberger, Benkert et al.).

Fungal symbioses among temperate bark and ambrosia beetles are already well-characterized, but 1,000's of representatives of these beetles in the tropics have been poorly investigated. Hence, it is not surprising that extensive surveys of bark beetles in tropical China revealed a high diversity of new *Geosmithia* fungal associates (Zhang et al.). Similarly, Jiang et al. described a new *Fusarium* symbiont, dominating fungal isolates from the mycangia of the ambrosia beetle *Euwallacea interjectus* in Japan. Typically, the mycangia modulate these mutualisms due to their selective properties, but this can be circumvented by artificial inoculation and co-culture of different fungal symbionts with the host beetles. Menocal et al. showed that beetles receiving native vs. heterospecific fungal mutualists may exhibit lower fitness when reared in the laboratory for five consecutive generations. This is an important result for applied research on these species and may explain why symbiont replacements are relatively common, especially when ambrosia beetles are introduced outside of their native range (Hulcr and Stelinski, 2017). On the other hand, this result should be interpreted with caution given that Menocal et al. performed the experiments using artificial media lacking plant-derived defensive compounds, which are known to strongly affect fungal growth in nature. Corroborating this, Diehl et al. showed that the fungal symbiont communities and their succession over time are very different when the ambrosia beetle *Xyleborinus saxesenii* was bred in artificial media vs. their natural substrate of beech wood.

From an applied perspective, the Research Topic presents interesting new discoveries on the attractiveness of fungal volatiles for bark and ambrosia beetles (Curculionidae: Scotylinae). First, a study on the European spruce bark beetle *Ips typographus* showed that volatiles of their ophiostomatoid fungal associates synergize aggregation pheromones that are used to overwhelm and mass-kill trees in this species (Jirošová et al.). Second, a paper on the invasive Asian ambrosia beetle *Xylosandrus germanus* resolved the proximate mechanisms underlying the mass-aggregating behavior of this species by demonstrating that this species solely relies on volatiles of their obligate fungal mutualists as aggregation signals (Gugliuzzo et al.). Importantly, these fungal volatiles may also attract these beetle species to heterospecific fungi, as shown by a study on *Ips typographus* again that was equally attracted to conspecific and heterospecific fungal associates of North American spruce bark beetles (Tanin et al.).

In conclusion, the Research Topic presents a nice overview of the current research coverage on the diversity of beetle-microbe symbiotic associations. In particular, it highlights the prevalence and importance of the external association with symbiotic fungi among not only ambrosia and bark beetles but also other wood-feeding beetle groups. On account of the enormous diversity of the Coleoptera, which contains over 400,000 described species and accounts for the majority of the macroscopic biodiversity in the terrestrial ecosystem, there is no doubt that many more interesting beetle-microbe symbiotic associations are still to be discovered and investigated, which comprise the research direction for future studies.

## Author contributions

All authors listed have made a substantial, direct, and intellectual contribution to the work and approved it for publication.
